# Definition of anatomical zero positions for assessing shoulder pose with 3D motion capture during bilateral abduction of the arms

**DOI:** 10.1186/s12891-015-0840-7

**Published:** 2015-12-09

**Authors:** Oliver Rettig, Britta Krautwurst, Michael W. Maier, Sebastian I. Wolf

**Affiliations:** Clinic for Orthopedics and Trauma Surgery, Heidelberg University Hospital, Schlierbacher Landstr. 200 a, 69118 Heidelberg, Germany

**Keywords:** Upper extremity, Kinematic modelling, Shoulder

## Abstract

**Background:**

Surgical interventions at the shoulder may alter function of the shoulder complex. Clinically, the outcome can be assessed by universal goniometry. Marker-based motion capture may not resemble these results due to differing angle definitions.

**Methods:**

The clinical inspection of bilateral arm abduction for assessing shoulder dysfunction is performed with a marker based 3D optical measurement method. An anatomical zero position of shoulder pose is proposed to determine absolute angles according to the Neutral-0-Method as used in orthopedic context. Static shoulder positions are documented simultaneously by 3D marker tracking and universal goniometry in 8 young and healthy volunteers. Repetitive bilateral arm abduction movements of at least 150° range of motion are monitored. Similarly a subject with gleno-humeral osteoarthritis is monitored for demonstrating the feasibility of the method and to illustrate possible shoulder dysfunction effects.

**Results:**

With mean differences of less than 2°, the proposed anatomical zero position results in good agreement between shoulder elevation/depression angles determined by 3D marker tracking and by universal goniometry in static positions. Lesser agreement is found for shoulder pro-/retraction with systematic deviations of up to 6°. In the bilateral arm abduction movements the volunteers perform a common and specific pattern in clavicula-thoracic and gleno-humeral motion with maximum shoulder angles of 32° elevation, 5° depression and 45° protraction, respectively, whereas retraction is hardly reached. Further, they all show relevant out of (frontal) plane motion with anteversion angles of 30° in overhead position (maximum abduction). With increasing arm anteversion the shoulder is increasingly retroverted, with a maximum of 20° retroversion. The subject with gleno-humeral osteoarthritis shows overall less shoulder abduction range of motion but with increased out-of-plane movement during abduction.

**Conclusions:**

The proposed anatomical zero definition for shoulder pose fills the missing link for determining absolute joint angles for shoulder elevation/depression and pro-/retraction. For elevation-/depression the accuracy suits clinical expectations very well with mean differences less than 2° and limits of agreement of 8.6° whereas for pro-/retraction the accuracy in individual cases may be inferior with limits of agreement of up to 24.6°. This has critically to be kept in mind when applying this concept to shoulder intervention studies.

## Background

Orthopedic interventions at the shoulder typically aim to prevent or resolve pain and restore or establish shoulder function with respect to range of motion (ROM) and strength. In osteoarthritic shoulders, function can be improved significantly by total shoulder arthroplasty, which is an established procedure [1]. Soft tissue procedures may improve function in rotator cuff tears [2]. Clinically, a typical functional test is to assess the patient’s active shoulder abduction aiming for a lateral overhead movement. The function then is documented via scores such as the Constant score [3] or via planar angle measurements with a manual goniometer (Neutral-0-Method [4]) quantifying typically the abduction of the arm relative to the thorax. Patients surgically treated for shoulder instability or rotator cuff tears show limited ROM in this movement and they often compensate with increased shoulder girdle and thorax motion [5]. More than 50 years ago planar measurements showed that about one third of the humerus-thoracic movements originate from the closed chain mechanism of thorax, clavicle and scapula (altogether the shoulder) and only two-thirds from gleno-humeral rotations [6].

In the last decade, various methods have been used to objectively analyze the complex arm and/or shoulder motion in vivo, using MRI [7], biplanar X-ray [8], noninvasive marker-less fluoroscopic imaging [9], 3D electromagnetic tracking [10], inertial sensing [11], ultrasound-based methods [12], and optical marker tracking. The last technique relies on kinematic models which relate positions of skin placed markers to joint kinematics. Up to now, the majority of the models are based on anthropometric reference data and linear regression for determining joint parameters [13, 14]. Other approaches make use of functional joint center and axes estimation for individual joint parameter determination [15] as also recommended by the International Shoulder Group of the International Society of Biomechanics (ISB) [16]. A major drawback in all skin- based methods, including sensor techniques, is that the orientation of the scapula and also rotations about the clavicle’s longitudinal axis are most difficult to access [17]. Some approaches estimate the orientation of the scapula in a limited ROM due to skin motion artefacts [11, 14, 18]. In the case of a marker based system this is called “acromion marker based method” [19, 20], whereas others reduce the description of shoulder kinematics to the two angles elevation/depression and pro-/retraction corresponding to the two rotational degrees of freedom (DoF) associated with the orientation of the clavicle’s long axis [21, 22]. In order to objectively assess shoulder function and, more specifically, shoulder pose and motion in the clinical context of orthopedic interventions by using a 3D marker tracking procedure, the following aspects should be considered: 1) feasibility, i.e., a small set of markers is quick to prepare, and disturb only little; 2) reliability and 3) validity in an adequate range of arm abduction angles and shoulder poses. The first two of these aspects are widely discussed in the literature for several measurement procedures and joint angles. However validity is difficult to prove because there is no gold standard available. In the clinical setting a seemingly intuitive assessment of planar angles in static shoulder poses is performed by universal goniometry. The Neutral-0-Method describes the examination details primarily for the large joints of the lower and upper extremity. This includes the definition of the plane in which the angle is measured and also the anatomical zero position in which the two adjacent segments typically are aligned in parallel (e.g. elbow flexion is zero when the elbow is fully extended and the forearm is aligned in parallel to the humerus). However, a rigorous definition for shoulder elevation/depression and pro-/retraction for shoulder elevation/depression and pro-/retraction is missing in this clinical context. Likewise 3D motion capture is lacking of a definition of a neutral shoulder position. Hence, the comparison of data obtained by universal goniometry and 3D motion capture for proving at least face validity in static cases may be misleading. The typical approach of 3D motion capture to quantify a static reference is not helpful since a “relaxed” position of the shoulder and shoulder girdle may not represent a zero position.

The aim of the presented work was therefore to establish an anatomical zero position of shoulder pose to be included in an objective 3D measurement procedure and to check face validity by universal goniometry in the determination of shoulder pose in a sequence of arm abduction and anteversion positions. This can be regarded as an confirmative test despite the well-known limitations of universal goniometry e.g. in repeatability and accuracy as discussed in [23].

The proposed anatomical zero definition is implemented as an extension to the Heidelberg Upper Extremity (HUX) model [24], to obtain absolute angles for shoulder elevation/depression and pro-/retraction and is represented by projection angles to directly be compared with angles obtained by universal manual goniometry. Projection angles have been used in preference to Euler/Cardan angles as they promise a more intuitive understanding and handling when taking into account anatomic offset angles.

Feasibility, reliability and face validity is tested in a group of young and healthy volunteers performing arm ab-/adduction movements. For monitoring effects of shoulder dysfunction, a subject with gleno-humeral osteoarthritis was assessed in the same manner as a sample case.

## Methods

### Subjects and measurement protocol

**Fig. 1:**
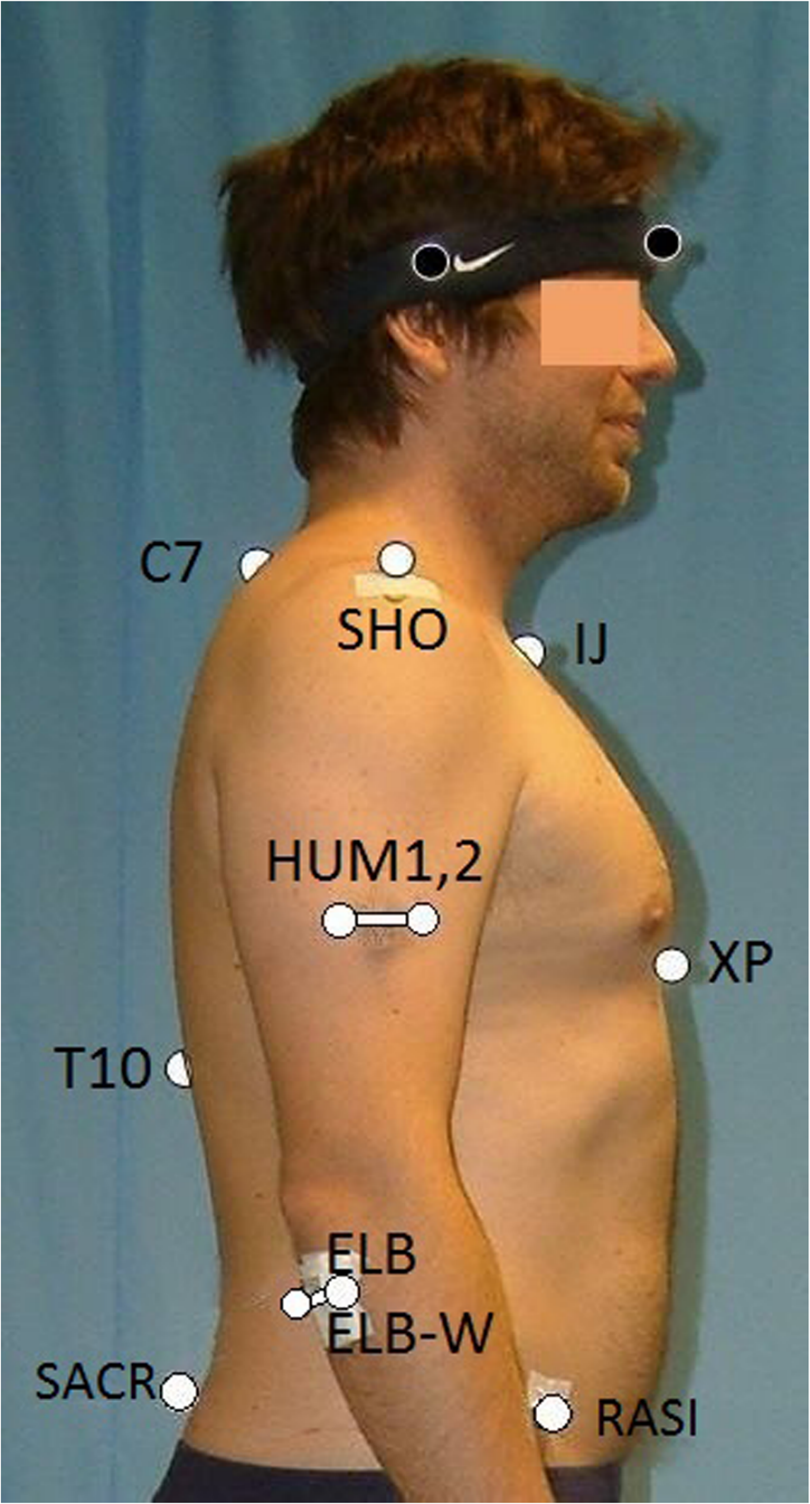
Marker placement: Thorax: IJ, XP, C7, T10; shoulder: SHO; humerus: twin marker HUM1, 2; elbow: ELB, ELBW; wrist: ULN, RAD (not shown); pelvis: LASI (not shown), RASI, SACR

Eight healthy volunteers (no movement limitations in the upper extremity, no upper back/neck hardening or pain, 29.1 ± 12.0 years, 4 male) and one 72 year old male subject with gleno-humeral osteoarthritis took part in the study. All tests for this study were conducted by a single examiner. In accordance with the World Medical Association Declaration, the study protocol was approved by the local ethics committee (S-305/2007), and written informed consent for participation in the study was obtained from all participants. Data were recorded using a 12 camera-Vicon 612 motion capture system with sample rate of 120Hz. As illustrated in Fig. 1 and listed in Appendix A, the following markers were applied: Four markers at the thorax: IJ, XP, C7, T10, one at the acromion: SHO, a twin marker at the humerus: HUM1, 2 (instead of only one marker as done in [25] a twin marker at the ulna distally to the olecranon: ELB, ELBW and two markers at the wrist: ULN, RAD (not shown in Fig. 1). Furthermore, three markers placed at the pelvis, namely, the left and right anterior superior iliac spine (LASI, RASI) and a dorsal marker placed midway between the posterior superior iliac spines (SACR).

For defining the upright thorax segment (Appendix E), marker positions were used from the subject in a standing position with thorax upright, the arms hanging loosely down, the fingers pointing to the floor and the thumbs pointing anteriorly, respectively (static calibration). For functional calibration of joint axes and centers [24], three independent dynamic trials were monitored bilaterally at least three times with thorax upright, each starting with the hanging arm. Arm ab-/adduction and ante-/retroversion movements were performed with the elbow in extension. Elbow flexion/extension movements were also performed with hanging arms, respectively. No further advice was given about how to position or move the shoulder in these calibration trials. They were used to determine the elbow joint axes which are needed for further joint angle calculation of the HUX model [24] only to reduce skin motion artifacts (see also Appendix G). If the HUX model is not used, there is no need for these calibration trials and for determination of the elbow joint axis, to define the proposed anatomical zero position. To monitor the maximum possible active ROM with respect to elevation/depression and pro-/retraction of the shoulder, a sequence of three repetitions of bilateral elevation/depression and pro-/retraction movements was performed by the subject with hanging arms and with the thorax in upright position. These trials are later referred to as “shoulder movement trials”.

**Fig. 2:**
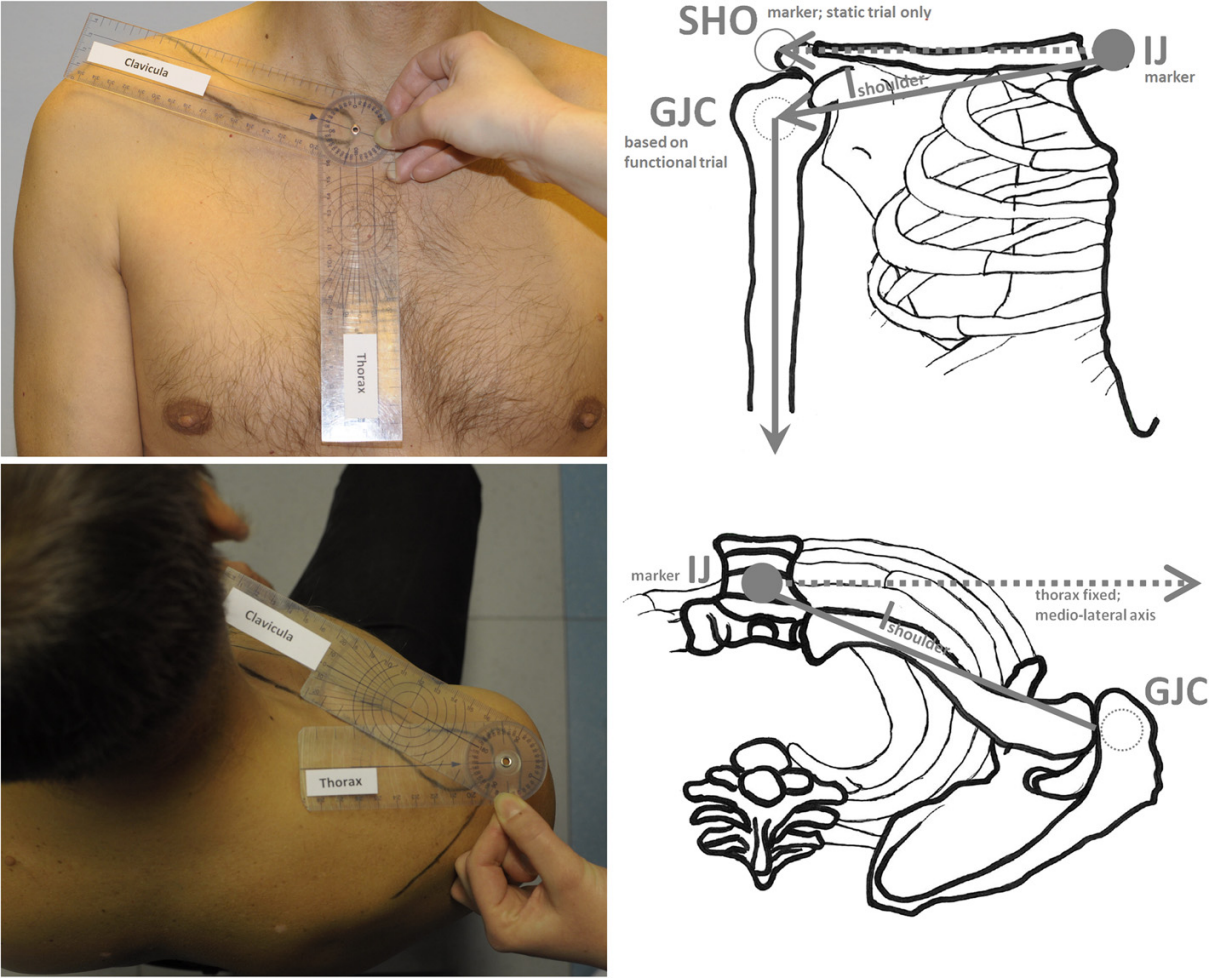
Shoulder elevation/depression measurement (top, left), Shoulder pro-/retraction measurement (bottom, left) with a prepared goniometer; Shoulder elevation neutral position: offset angle determination from static trial (top, right), Shoulder pro-/retraction neutral position: subject independent offset angle set of 20° in transverse plane

Additionally, six static shoulder positions were monitored simultaneously by universal goniometry and by 3D motion capture in all subjects. For these assessments, the subjects were asked to hold their shoulders”little”,”moderately”, and”extensively” elevated as well as”little”,”moderately”, and”extensively” protracted (“shoulder position trials”). These six shoulder positions, all with hanging arms, were thoroughly assessed with a universal goniometer (one degree increments) in the following way: Elevation was measured by placing the fulcrum of the goniometer in front of the sterno-clavicular joint, one arm of the goniometer being aligned with the vertical axis of the thorax and the other arm with the frontal perspective of the clavicle. Neutral position, i.e. zero degree elevation was then defined with the frontal perspective of the clavicle aligned perpendicular to the vertical axis of the thorax (compare Fig. 2, left, top). Protraction was measured by placing the fulcrum of a specifically prepared goniometer onto the acromion with the shortened arm directed along the medio-lateral axis of the thorax and the arm with rectangular cutout aligned with the transverse perspective of the clavicle (compare Fig. 2, left, bottom). Following the work by Culham et al. [26] neutral position with respect to shoulder protraction was defined as the alignment of the clavicle of 20° inclination in the transverse plane with respect to the medio-lateral axis of the thorax. Hence, angles smaller than 20° with respect to the medio-lateral axis indicate protraction and angles larger than 20° indicate retraction. It has to be stressed that the elevation and protraction angles determined in this manner for shoulder position relaxed as much as possible typically are not near zero. Further, in one subject the six static shoulder positions were tested eight times by the same examiner and also by four different examiners. Each test started from scratch: markers were re-applied and independent goniometer readings were performed. Altogether 192 cross-checks between simultaneously measurements by universal goniometry and 3D motion capture were performed for static positions.

### Modeling and angle conventions

The vertical axis of the thorax segment ($\overrightarrow{l} thorax$), is defined by static calibration between the center point of the three pelvis markers and the midpoint between C7 and the midpoint between LSHO and RSHO (Appendix B). In subsequent motion trials $\overrightarrow{l} thorax$ is determined by the technical coordinate system based exclusively on the four markers placed on the thorax as described in [24], (Appendix C). Shoulder motion is monitored based on a vector $\overrightarrow{l} shoulder$ (Appendix D) between the thorax marker *IJ* and the gleno-humeral joint center (GJC) determined by the functional calibration ([25, 27] as described in detail for the validated HUX model in [24]. This vector $\overrightarrow{l} shoulder$ is set fix medially at the thorax segment (Appendix E) and laterally at the humerus segment via ball-and-socket joints as done in [28]. Shoulder motion is then described with only two rotational degrees of freedom, namely, shoulder elevation/depression and pro-/retraction. These angles describe motion of the total shoulder complex rather than its bony parts even though they follow closely the motion of the clavicle as illustrated in Fig. 2.

Since $\overrightarrow{l} shoulder$ laterally points to the GJC and not directly to the acromion it is not aligned with the frontal aspect of the clavicle. Hence, a technical offset angle (Appendix F) is determined by static calibration for absolute shoulder elevation/depression angles Following the concept of the Neutral-0-Method, shoulder elevation/depression finally is defined as the angular sum of this technical shoulder elevation offset angle and the angle enclosed by $\overrightarrow{l} shoulder$ and the medio-lateral axis of the thorax projected into the frontal plane of the thorax (Appendix H.1).

Similarly pro-/retraction is defined as the angle enclosed by $\overrightarrow{l} shoulder$ and the medio-lateral axis of the thorax projected into the transverse plane of the thorax and subtracting an anatomic offset angle of 20° [26], as visualized in the bottom right picture of Fig. 2 (see also Appendix H.2). Note that for elevation/depression, the offset angle is purely technical in nature as the horizontal clavicle represents neutral shoulder elevation whereas for pro-/retraction the offset angle is due to the anatomic situation that in the neutral position of the shoulder girdle the lateral end of the clavicle is facing noticeably facing posteriorly, i.e. about 20°. Further, the position of the arm relative to the thorax is defined in an intuitive manner as described in Appendix I.

### Data analysis

The shoulder position trials with three different shoulder positions both for elevation and protraction are arranged into three groups: The intra-tester group consists of 24 measurements with the same subject taken by the same examiner with 8 repetitions. The inter-tester group consists of 12 measurements from 4 different testers repeating the complete measurement process. The inter-subject group consists of 24 measurements taken by the same examiner who measured the group of 8 subjects. Bland-Altmann diagrams [29] were created to assess the agreement between the two measurement methods. For statistical analysis, IBM SPSS Statistics Version 22 was used and single factor analyses of variance (ANOVA) were performed to test for differences between data groups.

For each subject’s dominant side, the active shoulder ROM with respect to elevation/depression and pro-/retraction was determined in shoulder movement trials via 3D marker tracking and averaged accordingly. Additionally the angles are determined for the following arm abduction positions: 0°, 30°, 60°, 90°, 120° and 150°.

For specifically monitoring shoulder motion and coordination, the repetitive arm ab-/adduction movements taken for gleno-humeral joint center calculation were separated into movement cycles and time normalized. Coefficients of multiple correlation (CMC) were determined as described in [30] without any offset adjustments.

Simple “reference bands” were calculated in the volunteer group assuming normal distribution for visual comparison [31]. Recent literature recommends more advanced statistics to establish prediction bands [32] instead but for computational ease and better comparison with previous literature this presentation was preferred.

## Results

In the shoulder position trials, the subjects chose to position their shoulders roughly between 15° and 25° both for shoulder elevation and protraction, with self-selected positions for “little” and “extensively” being typically only about 10° apart.

**Fig. 3:**
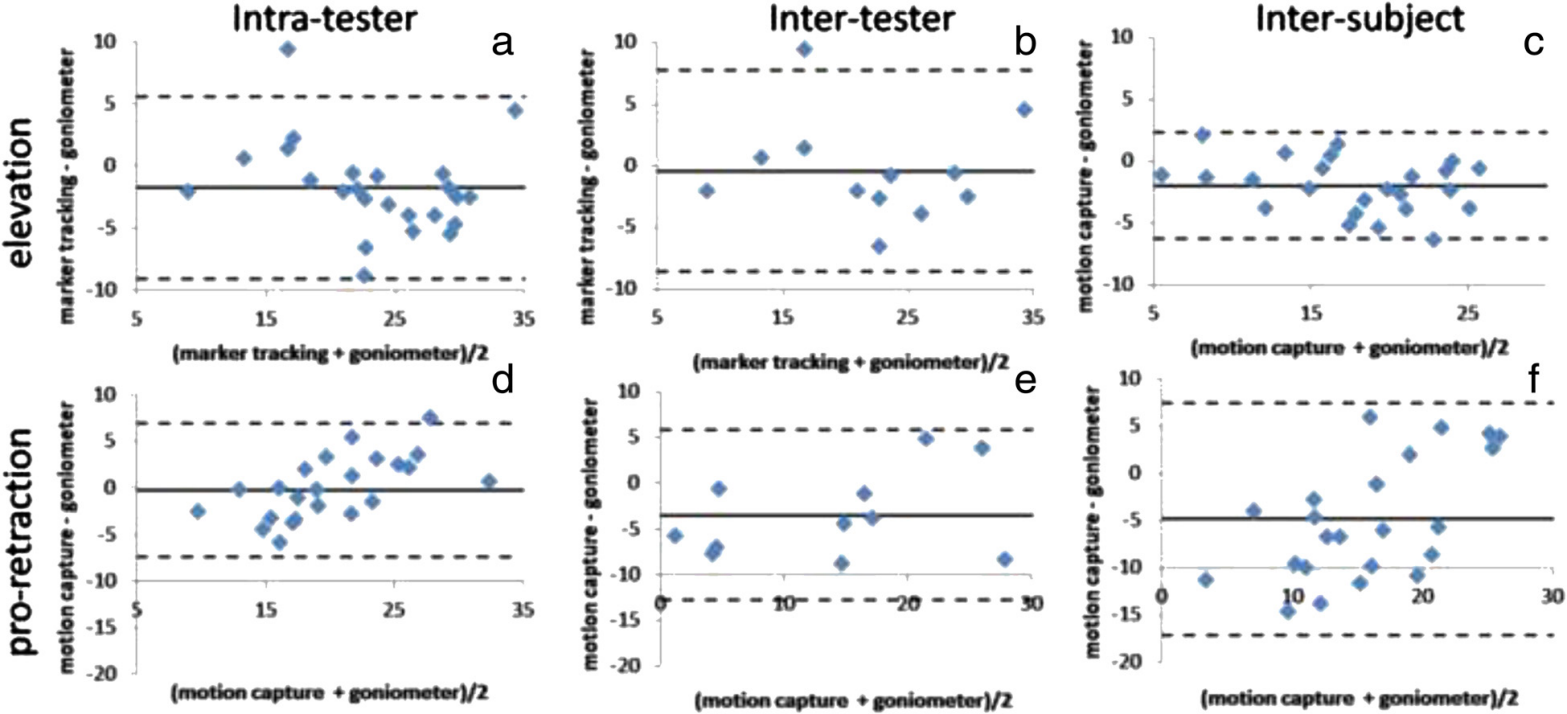
Bland-Altman diagrams: Data from eight healthy individuals; Goniometer and marker-based measurements of three static positions

The degree of agreement between measuring shoulder elevation/depression and pro-/retraction by 3D motion capture and by universal goniometry based on the described custom prepared goniometer is shown by Bland-Altman diagrams [29] in Fig. 3.

With differences between the groups mean values of less than 2° shown by the first three Bland-Altmann diagrams (a-c) the methods are in good agreement for shoulder elevation. The agreement is poorer for shoulder pro-/retraction (d-f) compared to elevation due to a negative shift with systematic differences of up to 5° to the inter-subject data group (f), where universal goniometry always determines larger protraction values than motion capture – a discrepancy which is not present in shoulder elevation measurements (a-c).

Intra-tester and inter-subject measurements of shoulder elevation (a,c) determined from the same tester show smaller SD (3.74°; 2.20°) and a negative shift in the mean differences between the methods (−1.74°;-1.98°) in comparison to the measurements from different testers (inter-tester (b): −0.42° ± 4.17°). Nevertheless, the differences in the mean for the three data groups (a-c) are probably random (*p* = 0.46).

Similar results are found for pro-/retraction for the intra-tester data group only (−0.21° ± 3.65°; (d)). Measurements from different testers (e) show a larger negative shift and a broader SD (−3.48° ± 4.76). Mean differences and SD of the measurements in the group of different subjects (inter-subject; (f)) show the biggest negative shift (−4.77°) and a still higher SD (6.26°). The differences in the mean values for the three data groups (d-f) are probably not random: F(2,56) = 5.053, *p* = 0.01.

In the shoulder movement trials, maximum shoulder protraction of 34.7° (SD 7.4°), maximum shoulder retraction of 8.3° (SD 4.3°), maximum shoulder elevation of 32° (SD 6°), and minimum shoulder elevation of 4.5° (SD 3.3)° was found on average in the reference group of eight healthy subjects. In the nomenclature of the Neutral-0-Method, these values correspond to a shoulder pro-/retraction ROM of (35|0|8); (max. pro-| 0| max. retraction) and to elevation/depression ROM of (32|5|0); (max. elev-| min. elevation| 0), indicating a “shoulder depression deficit” (no depressed position reached) found in all subjects.

**Fig. 4:**
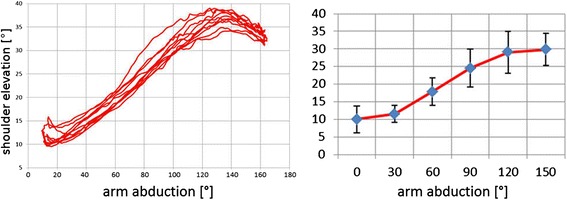
Left: Shoulder elevation during a sequence of 5 repetitions of ab-/adduction movements for the same subject. Right: Shoulder elevation during bilateral shoulder ab-/adduction; mean and SD of eight healthy subjects shown for six discrete shoulder abduction angles

A typical repetitive shoulder ab-/adduction pattern is illustrated in Fig. 4 (left), where shoulder elevation is plotted against arm abduction.

**Fig. 5:**
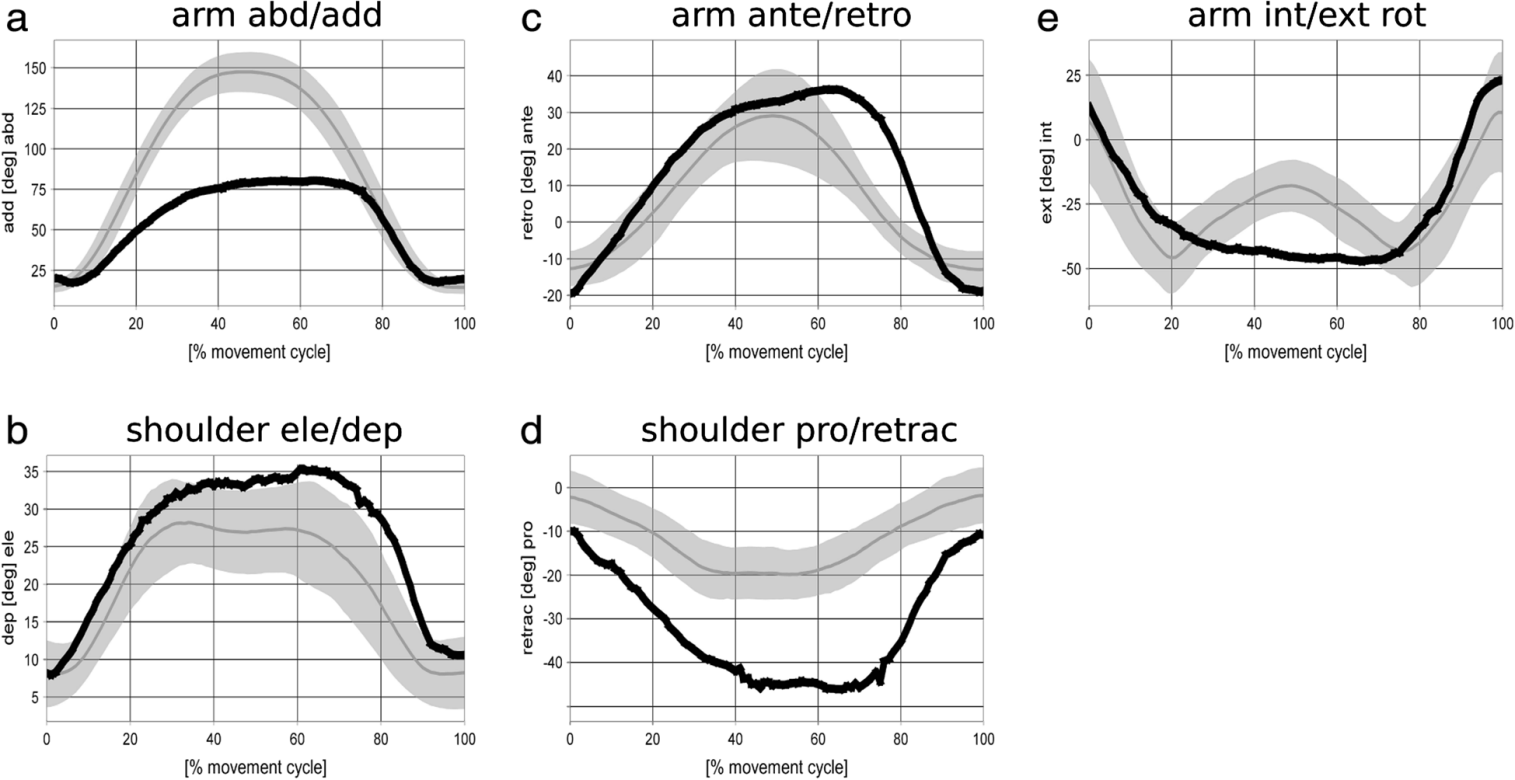
Motion graphs for bilateral arm ab-/adduction: Gray bands represent mean and SD for the reference group of 8 healthy subjects. Data of a sample patient with glenohumeral osteoarthritis are shown in black

The arm is abducted up to about 160°. Shoulder elevation increases with arm abduction and shows a characteristic maximum of 35° elevation at about 130° arm abduction. All healthy subjects abducted their arm by at least 150°. Inter-subject means for elevation are shown in 30° intervals in the range between 0 and 150° abduction in Fig. 4 (right). Overall this reference group shows little shoulder elevation in the first interval 0 - 30°, continuous increase in elevation in the interval 30 - 120°, and again little change in the interval 120 - 150° arm abduction. Characteristic maxima in shoulder elevation as illustrated in Fig. 5b were found in three out of eight subjects in whom arm abductions larger than 150° were observed.

The advised bilateral arm abduction movement was performed in a very similar and characteristic way by all volunteers, as reflected in the large CMC values (Table 1).

**Table 1:** Intra- and inter-subject repeatability for shoulder pro-/retraction and elevation, arm ante-/retroversion, ab-/adduction and int-/external rotation characterized by CMC values

	Shoulder		Arm		
	pro-/retraction	elevation	ante/retro	abduction	int/ext rot
intra-subject	0.95	0.97	0.95	0.99	0.87
inter-subject	0.68	0.87	0.74	0.97	0.66

In the time plots in Fig. 5, the gray bands illustrate in detail how the movement is performed. The arms are harmonically abducted with a maximum of typically 150° abduction at 50 % movement cycle (MC) (compare Fig. 5a), i.e., the arms are raised and lowered at the same speed. Maximum shoulder elevation is reached earlier, at about 30 % MC (compare Fig. 5b), and kept rather constant until 70 % MC with a slight dip at 50 % MC, i.e., at the position of maximum arm abduction. Furthermore, with increasing arm abduction the arms are continuously anteverted from typically 10° retroversion with hanging arms to about 30° anteversion at maximum abduction, i.e., at 50 % MC (compare Fig. 5c). Conversely, the shoulder moves into retraction, however, with a smaller ROM (pro-/retraction: (0|0|20); compare Fig. 5d). Throughout abduction until 90°, the arm is continuously externally rotated from 5° internal to 45° external rotation (compare Fig. 5e). For abduction angles larger than 90° the arm still rotates externally but the joint position is rated differently according to the concept of conjunct rotation [33].

In contrast to the healthy volunteers, the 72 year old patient with gleno-humeral osteoarthritis could only raise his arm up to 80° arm abduction (compared to 150° in the reference group; see Fig. 5a) but with increased shoulder elevation (35° compared to 30°; see Fig. 5b). Furthermore, the ROM in arm ante-/retroversion (40|0|20) is increased compared to (30|0|10); (see Fig. 5c) and shoulder pro-/retraction ROM (0|10|45) increased compared to (0|0|20); (see Fig. 5d). Similar to the reference subjects, the subject with gleno-humeral osteoarthritis continuously externally rotates his arm from 5° internal to 45° external rotation (see Fig. 5e).

## Discussion

Surgical interventions at the shoulder are performed as routine procedures to treat shoulder dysfunction of traumatic or degenerative causes. In recent years a number of in vivo methods have been developed to monitor motion of the shoulder complex. However, the direct comparison to the outcome assessed by universal goniometry is difficult. In the case of the scapula, there are well accepted standards in biomechanics to report its motion by Euler-Cardan angles but there is no standard in describing the motion of the shoulder as a whole [22]. In addition to the quasi 2D description of the shoulder in [28, 34] which describe the shoulder as a linear segment $\overrightarrow{l} shoulder$ mainly following the motion of the clavicle this work is focused to add the missing anatomical zero position definitions of the shoulder with respect to elevation and protraction. To define the required reference coordinate system of the thorax, markers placed on the pelvis are used to reduce artifacts based on individual differences mainly in the shape of the anterior thorax. The implementation is done as an extension of the HUX model [24] but the anatomical zero positions can also be used for any other measurement procedure. Despite its clinical importance, we know of only one publication by Nadeau and co-authors [35] taking an anatomical zero position for shoulder elevation into account but not for pro-/retraction. The specificity of this anatomical zero position definition is that it closely resembles the intuitive measurement process of planar angles at the shoulder complex with universal goniometry to tie in with the experiences of orthopedists.

The results of the shoulder position trials with respect to elevation (compare Fig. 3) show a comparatively good agreement between marker tracking and manual goniometer readings, with a mean difference less than 2° in the intra-tester and the inter-subject data group carried out from the same tester. In the inter-tester data set the mean difference is even less than 0.5°. This indicates that systematic differences are mainly influenced by the tester and not by the measured subject and in consequence should be regarded as a limitation of the universal goniometry. However this effect is not significant for the data groups presented. We are aware that manual goniometer readings cannot serve as a golden standard due to possible inaccuracies of 5° and more [23]. This matches with the limits of agreement in the Bland-Altmann diagrams. However, the cross-checking will at least confirm that data obtained by the optical method determine the same quantity compared to using a manual goniometer and hence prove at least face validity. The results of our model with respect to shoulder elevation are also confirmed in a dynamic situation by measurements with MRI [7] resembling the “shoulder rhythm” of clavicula-thoracic motion as illustrated in Fig. 5. However, in the MRI study shoulder elevation is found to be smaller by 4° throughout abduction which may be explained by the different definition of the anatomic zero position in our approach. In addition, measurements based on electromagnetic tracking of trans-cortical pins [36] showed the same pattern but with less ROM which may be explained by the fact that sterno-clavicular motion in a standing position instead of thoracic-clavicular motion was measured. To our knowledge, the only other study using optical motion capture with a similar approach to monitor shoulder elevation during arm abduction is the work by Garofalo et al. [22]. Their results showed a similar ROM in elevation but shoulder abduction was only monitored up to 120° and no explicit anatomical zero position was taken into account at all.

With respect to protraction, the agreement between marker tracking and manual goniometer readings in the shoulder position trials is weaker compared to the results obtained for shoulder elevation. The inter-subject data group shows a much larger systematic difference (−6.99°) than the inter-tester data group (−3.48°). Significant differences between the three data groups are found (*p* = 0.0064) indicating that the agreement between the measurement methods strongly depends on subject specifics. Accordingly the limits of agreement are broader. With 24.6° as the largest value in the inter-subject data group, this value is much weaker for protraction compared to 8.6° in elevation which meets the clinical rule of thumb expectation that manual goniometer measurements give an accuracy of about 5–10°.

The fact that for shoulder protraction we do not obtain equally satisfying results as for shoulder elevation, may be a consequence of the limitation that clavicle motion is modeled by only two instead of three DoF or simply reflect the situation that the clinical examiner can assess frontal plane (facing) angles more easily compared to assessing the persons shoulder position from above. However, “Pitching” of the shoulder, i.e. rotation about the long axis of the clavicle cannot be monitored by this model approach (and neither by universal goniometry), which does not take any information of the scapula into account. Hence shoulder motion can involve subject specific relevant shoulder pitching and may therefore influence the data on shoulder protraction. Similarly, the goniometer measurements are more difficult to be performed for shoulder pro-/retraction than for elevation because the alignment of the goniometer to the medio-lateral reference must rely on a much shorter lever arm.

The shoulder function primarily monitored in this work with lateral overhead movements of both arms, was proven to be performed in a very specific and repetitive pattern quantified by large CMC values (compare Table 1) and including out-of-plane motion for shoulder and arm (compare Fig. 5). The definition of the anatomical zero position allows to directly compare individual resting positions of the shoulder as well as maximum shoulder depression and elevation angles. None of the subjects studied in this work was able to actively depress (i.e. lower) the shoulder beneath anatomical zero position. All of the subjects have an individual but always elevated shoulder in their most relaxed position. The measured value of 5° agrees with the value of 5.9 ± 1° for the bony clavicle position given in [36].

To demonstrate the feasibility of the presented method in a clinical context and to illustrate possible shoulder dysfunction effects, a 72-year old man with gleno-humeral osteoarthritis was also monitored in the same manner as the young group of volunteers. The test took about 30 min with this very compliant subject reporting no negative effects. He showed overall less ROM in arm abduction which is a typical finding in these subjects. In contrast, when taking into account this small ROM, in relation he showed noticeably larger shoulder elevation motion. Further, he demonstrated an increased out-of-plane movement i.e. the arm performed also ante-/retroversion of 40° and the shoulder performed also a pro-/retraction of 35° during this lateral arm overhead reach. These findings may be explained as compensation mechanisms in order to reduce motion of the degenerated gleno-humeral joint. However, as the reference group is much younger this reasoning is preliminary. Also age dependent reduced proprioception as found in [37] may be responsible for increased out-of-plane motion. Nevertheless, the clear difference in pattern found in this subject in relation to the reference group gives rise to the hope that further studies using this marker-based model for monitoring degenerative or traumatic shoulder dysfunction will give more insight into biomechanical and functional changes induced by surgical interventions at the shoulder.

### Limitations

Only a small number of healthy young volunteers have participated on the study. For analyzing the definition of the anatomical zero position and in particular to prove correspondence with universal goniometry at the level of face validity this is sufficient. However, with respect to intended applications in rather elderly subjects clearly other reference data will have to be taken and results presented in this context here are preliminary.

Furthermore, marker based measurements with humerus elevation more than 150° are critical. For the HUX model it is shown that ad-/abduction and ante-/retroversion angles agrees very good with universal goniometry up to 120° and above differences stay less than 10°. Face-validity for shoulder elevation and pro-/retraction is shown in this study for the hanging arm only. However, we expect that the absolute shoulder angles are also well defined for the more elevated arm because the GJC in the HUX model is determined as a function of the positions of markers placed on the arms only. In any case, it is a limitation of the study that no explicit tests were done for more elevated arm positions.

## Conclusions

The missing link to determine absolute angles is filled by the proposed feature of an anatomical zero position definition. This allows for determining absolute joint angles independent from a static reference position. The feature was implemented successfully as an extension to the HUX model [24] to determine absolute shoulder elevation/depression and pro-/retraction angles. The definition of zero positions on the basis of correspondence with universal goniometry for static positions facilitates to create confidence in the method by orthopedists because the angles definitions are known and hence self-explanatory. Hence, the method is suitable for objectively and quantitatively assessing shoulder dysfunction in frontal plane movements as illustrated for eight volunteers and in the case with gleno-humeral osteoarthritis. In contrast, shoulder pro-/retraction shows systematic differences between the different data groups (inter-tester, intra-tester, inter-subject) for the mean differences between marker-based angle determination and manual goniometer measurements. Accuracy in sagittal plane shoulder motion may be lower for specific subjects.

The strategy to define anatomical zero positions in correspondence with universal goniometry has been found very helpful despite its obvious limitations to static measurements and its low accuracy.

## Appendix

### Apendix A: Markers

**Table:**

**marker**	**Location**	**segment**
IJ	deepest point of incisura jugularis	thorax
XP	xiphoid process,	thorax
C7	spinous process of C7	thorax
T10	spinous process of T10	thorax
SHO	on top of acromion	shoulder girdle
ELB-ELBW	Left and right ulna distally to the olecranon	forearm/ulna
ULN	Near left and right processus styloideus of ulnae and radii to represent best the hand flexion axis	forearm/ulna
RAD		forearm/radius
HUM-HUMW	left and right tuberositas deltoidea humeri	humerus
SACR	posterior superior iliac spine	pelvis
ASI	left and right anterior superior iliac spine	pelvis

### Appendix B: Thorax vertical axis

$$ \overrightarrow{l} thorax(t)=\frac{1}{4}\left( LSHO+ RSHO\right)+\frac{1}{2}C7-\frac{1}{3}\left( SACR+ LASI+ RASI\right) $$

- determined from a specific trial for static calibration, then fixed to the thorax TF

### Appendix C: Thorax TF

$$ {\varSigma}_{thorax}(t)=\left\{\overrightarrow{o} thorax(t),\overrightarrow{x} thorax(t),\overrightarrow{y} throax(t),\overrightarrow{z} thorax(t)\right\} $$

with:

$$ \begin{array}{l}\overrightarrow{o} thorax=\frac{1}{2}\left(IJ+C7\right)\\ {}\overrightarrow{x} thorax(t)=\frac{IJ-C7}{\left|IJ-C7\right|}\\ {}\overrightarrow{y} thorax(t)=\frac{\left(\left(IJ+C7\right)-\left(XP+T10\right)\right)}{\left|\left(\left(IJ+C7\right)-\left(XP+T10\right)\right)\right|}\times \overrightarrow{x} thorax(t)\\ {}\overrightarrow{z} thorax(t)=\overrightarrow{x} thorax(t)\times \overrightarrow{y} throax(t)\\ {}\end{array} $$

### Appendix D: Shoulder vector

$$ \overrightarrow{l} shoulder(t)=GJC-IJ+\frac{IJ-C7}{\left|IJ-C7\right|}\frac{d}{2} $$

The last term is an offset term to take the marker diameter (d) into account.

### Appendix E: Thorax AF

$$ {\varSigma}_{thoraxA}(t)=\left\{\overrightarrow{o} thorax(t),\overrightarrow{x} thorax A(t),\overrightarrow{y} throaxA(t),\overrightarrow{z} thorax A(t)\right\} $$

with:

$$ \begin{array}{l}\overrightarrow{z} thorax A(t)=\overrightarrow{x} thorax(t)\\ {}\overrightarrow{y} thorax A(t)=\overrightarrow{l} thorax(t)\\ {}\overrightarrow{x} thorax A(t)=\overrightarrow{y} thorax A(t)\times \overrightarrow{z} throaxA(t)\end{array} $$

### Appendix F: Shoulder elevation offset angle

$$ o= \arccos \left(\frac{\left|SHO-IJ\right|}{\left|SHO-GJC\right|}\right) $$

- determined from a specific trial for static calibration

### Appendix G: Elbow joint axis and elbow joint center

The elbow joint axis is determined by functional method [27] and an “elbow joint center” is defined as the point of the elbow joint axis with has minimal distance to GJC. If the calibration procedure is done, the point can be determined from markers placed on the forearm only. In other words, the elbow joint axis and the elbow joint center is needed only for the HUX-models specific GJC determination.

### Appendix H: Shoulder orientation against the thorax

1. Shoulder-elevation defined as the angle between $\overrightarrow{l} shoulder$ and $\overrightarrow{l} thorax$ minus 90° and plus the shoulder elevation offset angle.
2. Shoulder pro-/retraction is defined as the angle inclosed by $\overrightarrow{l} thorax$ and the projections of $\overrightarrow{l} shoulder$ into the transverse plane (defined by $\overrightarrow{l} thorax$ its normal vector) minus 30°.

### Appendix I: Arm orientation against the thorax

1. Arm abduction defined between the longitudinal axis $\overrightarrow{l} thorax$ of the thorax pointing down and $\overrightarrow{l} humerus$.
2. Arm ante-/retroversion defined as the angle between the axis pointing to posterior and $\overrightarrow{l} humerus$ minus 90°.
3. Arm int-/external-rotation is defined following [33].

## Abbreviations

ROM: range of motion
MRI: magnetic resonance imaging
ISB: International Society of Biomechanics
DoF: degrees of freedom
3D: three-dimensional
HUX: upper extremity model
GJC: Gleno-humeral joint center
CMC: coefficients of multiple correlation
SD: standard deviation
MC: movement cycle

## References

[1] Deshmukh AV, Koris M, Zurakowski D, Thornhill TS (2005). Total shoulder arthroplasty: long-term survivorship, functional outcome, and quality of life. J Shoulder Elb Surg.

[2] Omid R, Lee B (2013). Tendon transfers for irreparable rotator cuff tears. J Am Acad Orthop Surg.

[3] Constant CR, Murley AH (1987). A clinical method of functional assessment of the shoulder. Clin Orthop Relat Res.

[4] Gerhardt JJ (1983). Clinical measurements of joint motion and position in the neutral-zero method and SFTR recording: basic principles. Int Rehabil Med.

[5] Ludewig PM, Cook TM (2000). Alterations in shoulder kinematics and associated muscle activity in people with symptoms of shoulder impingement. Phys Ther.

[6] Freedman L, Munro RR (1966). Abduction of the arm in the scapular plane: scapular and glenohumeral movements. A roentgenographic study. J Bone Joint Surg Am.

[7] Sahara W, Sugamoto K, Murai M, Yoshikawa H (2007). Three-dimensional clavicular and acromioclavicular rotations during arm abduction using vertically open MRI. J Orthop Res.

[8] Bey MJ, Kline SK, Zauel R, Lock TR, Kolowich PA (2008). Measuring dynamic in-vivo glenohumeral joint kinematics: technique and preliminary results. J Biomech.

[9] Massimini DF, Warner JJ, Li G (2011). Non-invasive determination of coupled motion of the scapula and humerus--an in-vitro validation. J Biomech.

[10] Lin JJ, Hanten WP, Olson SL, Roddey TS, Soto-Quijano DA, Lim HK (2005). Functional activities characteristics of shoulder complex movements: Exploration with a 3-D electromagnetic measurement system. J Rehabil Res Dev.

[11] Parel I, Cutti AG, Fiumana G, Porcellini G, Verni G, Accardo AP (2012). Ambulatory measurement of the scapulohumeral rhythm: intra- and inter-operator agreement of a protocol based on inertial and magnetic sensors. Gait Posture.

[12] Illyes A, Kiss RM (2007). Shoulder joint kinematics during elevation measured by ultrasound-based measuring system. J Electromyogr Kinesiol.

[13] Butler EE, Ladd AL, Louie SA, Lamont LE, Wong W, Rose J (2010). Three-dimensional kinematics of the upper limb during a Reach and Grasp Cycle for children. Gait Posture.

[14] van Andel CJ, Wolterbeek N, Doorenbosch CA, Veeger DH, Harlaar J (2008). Complete 3D kinematics of upper extremity functional tasks. Gait Posture.

[15] Jackson M, Michaud B, Tetreault P, Begon M (2012). Improvements in measuring shoulder joint kinematics. J Biomech.

[16] Wu G, van der Helm FC, Veeger HE, Makhsous M, Van Roy P, Anglin C (2005). ISB recommendation on definitions of joint coordinate systems of various joints for the reporting of human joint motion--Part II: shoulder, elbow, wrist and hand. J Biomech.

[17] Brochard S, Lempereur M, Remy-Neris O (2011). Double calibration: an accurate, reliable and easy-to-use method for 3D scapular motion analysis. J Biomech.

[18] Meskers CG, van de Sande MA, de Groot JH (2007). Comparison between tripod and skin-fixed recording of scapular motion. J Biomech.

[19] van Andel C, van Hutten K, Eversdijk M, Veeger D, Harlaar J (2009). Recording scapular motion using an acromion marker cluster. Gait Posture.

[20] Warner MB, Chappell PH, Stokes MJ (2012). Measuring scapular kinematics during arm lowering using the acromion marker cluster. Hum Mov Sci.

[21] Klopcar N, Lenarcic J (2006). Bilateral and unilateral shoulder girdle kinematics during humeral elevation. Clin Biomech.

[22] Garofalo P, Cutti AG, Filippi MV, Cavazza S, Ferrari A, Cappello A (2009). Inter-operator reliability and prediction bands of a novel protocol to measure the coordinated movements of shoulder-girdle and humerus in clinical settings. Med Biol Eng Comput.

[23] DE Heelbrandt FA, Moore ML (1949). The measurement of joint motion: Part III - Reliability of Goniometry. Phys Ther Rev.

[24] Rettig O, Fradet L, Kasten P, Raiss P, Wolf SI (2009). A new kinematic model of the upper extremity based on functional joint parameter determination for shoulder and elbow. Gait Posture.

[25] Kasten P, Maier M, Rettig O, Raiss P, Wolf S, Loew M (2009). Proprioception in total, hemi- and reverse shoulder arthroplasty in 3D motion analyses: a prospective study. Int Orthop.

[26] Culham E, Peat M (1993). Functional anatomy of the shoulder complex. J Orthop Sports Phys Ther.

[27] Gamage SS, Lasenby J (2002). New least squares solutions for estimating the average centre of rotation and the axis of rotation. J Biomech.

[28] Klopcar N, Tomsic M, Lenarcic J (2007). A kinematic model of the shoulder complex to evaluate the arm-reachable workspace. J Biomech.

[29] Bland JM, Altman DG (1986). Statistical methods for assessing agreement between two methods of clinical measurement. Lancet.

[30] Kadaba MP, Ramakrishnan HK, Wootten ME, Gainey J, Gorton G, Cochran GV (1989). Repeatability of kinematic, kinetic, and electromyographic data in normal adult gait. J Orthop Res.

[31] Duhamel A, Bourriez JL, Devos P, Krystkowiak P, Destee A, Derambure P (2004). Statistical tools for clinical gait analysis. Gait Posture.

[32] Cutti AG, Parel I, Raggi M, Petracci E, Pellegrini A, Accardo AP (2014). Prediction bands and intervals for the scapulo-humeral coordination based on the Bootstrap and two Gaussian methods. J Biomech.

[33] Wolf SI, Fradet L, Rettig O (2009). Conjunct rotation: Codman’s paradox revisited. Med Biol Eng Comput.

[34] Klopcar N, Lenarcic J (2006). Bilateral and unilateral shoulder girdle kinematics during humeral elevation. Clin Biomech.

[35] Nadeau S, McFadyen BJ, Malouin F (2003). Frontal and sagittal plane analyses of the stair climbing task in healthy adults aged over 40 years: what are the challenges compared to level walking?. Clin Biomech (Bristol, Avon).

[36] Ludewig PM, Phadke V, Braman JP, Hassett DR, Cieminski CJ, LaPrade RF (2009). Motion of the shoulder complex during multiplanar humeral elevation. J Bone Joint Surg Am.

[37] Maier MW, Niklasch M, Dreher T, Wolf SI, Zeifang F, Loew M (2012). Proprioception 3 years after shoulder arthroplasty in 3D motion analysis: a prospective study. Arch Orthop Trauma Surg.

